# Crustacean Female Sex Hormone From the Mud Crab *Scylla paramamosain* Is Highly Expressed in Prepubertal Males and Inhibits the Development of Androgenic Gland

**DOI:** 10.3389/fphys.2018.00924

**Published:** 2018-07-17

**Authors:** An Liu, Jing Liu, Fang Liu, Yiyue Huang, Guizhong Wang, Haihui Ye

**Affiliations:** ^1^College of Ocean and Earth Sciences, Xiamen University, Xiamen, China; ^2^Fujian Collaborative Innovation Center for Exploitation and Utilization of Marine Biological Resources, Xiamen, China

**Keywords:** crustacean female sex hormone, eyestalk ganglion, androgenic gland, sex differentiation, mud crab

## Abstract

Recently, the crustacean female sex hormone (CFSH), which is considered a female-specific hormone, has been shown to play a crucial role in female phenotypes in crustaceans. In this study, two transcripts (*Sp-CFSH1* and *Sp-CFSH2*) encoding the same CFSH precursor were cloned from the mud crab *Scylla paramamosain*. Homology and phylogenetic analysis showed that CFSHs were homologous to interleukin-17 and highly conserved among brachyuran crabs. PCR analysis revealed that *Sp-CFSH* was expressed exclusively in the eyestalk ganglion of both prepubertal males and females, and surprisingly, the abundance of *Sp-CFSH* transcripts detected in the males were not significantly different from that of the females (*P* > 0.05). In addition, mRNA *in situ* hybridization showed that *Sp-CFSH* was localized in the X-organ of the male eyestalk ganglion. During the development of the androgenic gland (AG), the level of *Sp-IAG* mRNA in AG remained at low levels from stages I to II (early stage) but had a significant increase at stage III (mature stage). In contrast, the level of *Sp-CFSH* transcripts in the eyestalk ganglion was high in the early stage but extremely low in the mature stage. To investigate the potential function of CFSH in male *S. paramamosain*, the recombinant protein (∼20 kDa) was expressed in *Escherichia coli* and was subsequently added to AG explants *in vitro*. It was demonstrated that recombinant *Sp-CFSH* protein significantly reduced the expression of *Sp-IAG* in the AG explants at a concentration of 10^−6^ M (*P <* 0.05). In conclusion, our study provides the first piece of evidence that shows CFSH from the eyestalk ganglion acts as a negative regulator inhibiting the development of AG in crustaceans.

## Introduction

In the animal kingdom, most species have separate genders, which often can be distinguished by different sexual characteristics relating to male and female in gonads, the genital tract, or the external genitalia (Nagaraju, 2011). Under the control of chromosome composition and/or other factors, the primitive gonad in the embryo differentiates into testis or ovary, and the secondary male or female characteristics gradually appear with ontogenetic development (Hughes, 2001; Campos-Ramos et al., 2006). For brachyuran crabs, gender could be identified based on the secondary sexual features at the juvenile crab stage, such as the differentiations in the gonopore, pleopods, and abdominal shape (Lee et al., 1994; Nagaraju, 2011). For example, in the crab *Eriocheir japonicus*, the first pair of pleopods in males starts to appear at the third juvenile crab stage, but the third to fifth pleopods degenerate in males, while the second to fifth pleopods continue to develop and branch into two podites in females at the fourth juvenile crab stage (Lee et al., 1994).

In crustaceans, male sex differentiation is largely controlled by the androgenic gland (AG), an organ unique to males (Charniaux-Cotton, 1992). The function of this gland was first reported in the amphipod *Orchestia gammarellus* (Charniaux-Cotton, 1954). Thereafter, the androgenic gland hormone (AGH), also termed AG-specific insulin-like peptide (IAG), and the cDNA of IAG were isolated and characterized from the terrestrial isopod *Armadillidium vulgare* (Okuno et al., 1997, 1999). By removing AG or by the silencing of IAG gene from males, as well as by AG implantation or injection of AG extracts into females, the influence of AG/IAG on primary and secondary sex characteristics has been verified in various crustacean species (reviewed by Khalaila et al., 2001; Sagi and Khalaila, 2001; Barki et al., 2003; Manor et al., 2004; Rosen et al., 2010; Ventura et al., 2011b). For instance, in the giant freshwater prawn *Macrobrachium rosenbergii*, removing AG from juvenile males resulted in sex reversal (Sagi et al., 1997). Furthermore, silencing of the *Mr-IAG* led to the hypertrophy of AG and the arrest of testicular spermatogenesis (Ventura et al., 2009). It has also been shown that the expression of *Mr-IAG* could be detected at the postlarval stage; during the subsequent development, the pubertal males still required IAG to develop into reproductive-competent adult males (Ventura et al., 2011a).

In crustacean males, embryonic gonads develop into testes under the control of AG; on the other hand, embryonic gonads develop into ovaries in females without the influence of a well-defined equivalent gland (Katakura, 1989). However, the mechanisms underlying the regulation of female sex differentiation and sexual characteristics maintenance remain unclear. Recently, Zmora and Chung (2013) isolated a female specific hormone, named crustacean female sex hormone (CFSH), from the eyestalk of the female blue crab (*Callinectes sapidus*) and demonstrated that it played a crucial role in the development of female reproductive phenotypes. It was shown that the silencing of *CFSH* or bilateral eyestalk ablation impaired the mating and maternal care structures of females, such as absent or misplaced gonopores, sharper abdomens, as well as shorter and fewer setae on pleopods (Zmora and Chung, 2013). However, the absence of CFSH neither resulted in ovarian degeneration nor sex reversal; therefore, CFSH appears to be responsible for sex differentiation rather than sex determination (Zmora and Chung, 2013; Adkins-Regan, 2014). The discovery of CFSH suggested that AGH was not the sole sex differentiation hormone in crustaceans, and like males, female crustaceans may also require a feminizing hormone to reach sexual maturity (Adkins-Regan, 2014).

The endocrine axis of the eyestalk – AG – testis has been established in male crustaceans, in which AG has been shown to be regulated by the negative control of the eyestalk (Khalaila et al., 2002). For example, in the spot shrimp *Pandalus platyceros* and the swimming crab *Portunus pelagicus*, the hypertrophic AG and the increased numbers of IAG-producing cells were observed in males that had bilateral eyestalks removed (Hoffman, 1968; Sroyraya et al., 2010). Likewise, in the mud crab *Scylla paramamosain* and the blue crab *C. sapidus*, eyestalk ablation resulted in the up-regulation of *IAG* expression in AG (Chung et al., 2011; Zhang et al., 2014). These previous studies all demonstrated that the activity of AG was inhibited by the specific substance(s) derived from the eyestalk; however, the exact mechanism(s) responsible for this inhibition is still unknown (Khalaila et al., 2002).

To date, only a few CFSHs have been isolated from crustacean species, and little is known about the functions of this newly discovered hormone. In *C. sapidus*, CFSH was considered as a female-specific hormone, although CFSH was also detected in the eyestalk of the mature male *C. sapidus* at extremely low levels (Zmora and Chung, 2013). The presence of CSFH in the male eyestalk may indicate that CFSH also has a role to play in males. It is therefore interesting to explore whether CFSH is also present in males of other crab species. If yes, could it also be involved in regulating male sex differentiation, governed principally by AG/IAG.

In this study, we investigated a putative function of CFSH in male mud crab *S. paramamosain*. We firstly cloned two transcripts of *Sp-CFSH* from the eyestalk ganglion of *S. paramamosain* and found that *Sp-CFSH* had high expression in prepubertal males. Based on the expression profiles of *Sp-CFSH* and *Sp-IAG* during the development of AG, we hypothesized that CFSH might act as an inhibitory factor suppressing the development of AG. To verify this hypothesis, we cloned and purified the recombinant *Sp-CFSH* protein (rCFSH) from the prokaryotic expression system and subsequently added it into AG explants to vindicate the role of CFSH in inhibiting *Sp-IAG* expression.

## Materials and Methods

### Animals

The mad crabs (*S. paramamosain*) used for the experiments were obtained from a local fish market in Xiamen, Fujian, China. They were maintained in rectangular tanks with seawater at a temperature of 27 ± 2°C and a salinity of 28 ± 0.5 ppt for a week, during which they were fed with the clam *Ruditapes philippinarum*. Before tissue dissection, prepubertal crabs (females: carapace width 6.8 ± 0.5 cm, body weight 103.2 ± 4.3 g; males: carapace width 7.4 ± 0.5 cm, body weight 110.3 ± 3.2 g) at intermolt stage were placed on ice for anesthetization (*n* = 6). Various tissues including eyestalk ganglion, thoracic ganglion, cerebral ganglion, hepatopancreas, hemocytes, muscle, heart, stomach, gill, epidermis, Y-organ, mandibular organ, and gonad (ovary or testis) were then collected for tissue distribution analysis.

The development of AG in *S. paramamosain* can be classified into four stages (Ye et al., 2003; Liu et al., 2008): At stage I, the gland is short and the glandular cells are of small amounts. At stage II, the gland distinctively takes on a cord-figure. At stage III, the volume of the gland reaches its peak, with hyperplasia in some portion. At stage IV, the gland no longer grows and degenerate rapidly. Eyestalk ganglia and AGs from male crabs (*n* = 6) at stage I (carapace width 6.1 ± 0.5 cm, body weight 45.2 ± 4.3 g), stage II (carapace width 9.3 ± 0.5 cm, body weight 145.6 ± 10.5 g), and stage III (carapace width 14.3 ± 0.5 cm, body weight 245.8 ± 12.5 g) were collected for *Sp-CFSH* and *Sp-IAG* expression (GenBank accession number: KJ870255) profiles analysis. Tissues were immediately frozen in liquid nitrogen, and stored at −80°C until total RNA extraction.

### Full-Length Cloning of *Sp-CFSH*

Total RNA was extracted from the eyestalk ganglion of a mature female via TRIzol^®^ reagent (Invitrogen). The first-strand cDNA was generated with 1 μg total RNA using RevertAid First Strand cDNA Synthesis Kit (Fermentas). Degenerate primers CFSH-DF1 and CFSH-DR1 were designed based on the CFSH sequences of *C. sapidus* (GenBank accession number: GU016328.1) and *Carcinus maenas* (GenBank accession number: HM594946.1). The PCR reaction was performed in the ABI 2720 Thermal Cycler (Applied Biosystems) with Ex-Taq polymerase (Takara) under the following conditions: 95°C for 3 min; 35 cycles of 95°C for 30 s, 58°C for 30 s and 72°C for 50 s, followed by 72°C for 10 min final extension. After the amplification, the products were visually examined by 1.0% agarose gel. Subsequently, the fragment of *Sp-CFSH* was purified and then inserted into PMD19-T vector (Takara) for sequencing.

To obtain the full length cDNA of *Sp-CFSH*, 5′ and 3′ rapid amplification cDNA ends (RACE) were performed with SMART^TM^ RACE cDNA Amplification Kit (Clontech) according to the manufacturer’s instructions. The 5′-RACE cDNA template was first amplified with primers CFSH5R1 and the universal primer, and then a nested PCR was performed with the dilute amplicon by primer CFSH5R2 and the short universal primer in the same conditions. The 3′-ends of the sequences were obtained by using 3′-RACE cDNA with universal primer and CFSH3F1, and nested amplification with nested universal primer and CFSH3F2. The two rounds of 3′-RACE-PCR were performed following the conditions of 5′-RACE-PCR. All PCR products were extracted using a 1.0% agarose gel and then subjected to sequence as described above. Finally, the sequences were assembled and its homology was examined using the Blast *N.* The primers were all listed in Supplementary Table 1.

### Homology and Phylogenetic Analysis

The open reading frame (ORF) was predicted by the ORF finder^1^. The signal peptide was predicted by Signal P4.0^2^. The deduced CFSH amino acid sequences of crustacean species were arranged using Clustal X software. The phylogenetic tree was constructed by the maximum likelihood method with MEGA6 software.

### Tissue Distribution of *Sp-CFSH* in Males and Females

Total RNA was extracted from eyestalk ganglion and other tissues (including thoracic ganglion, cerebral ganglion, hepatopancreas, hemocytes, muscle, heart, epidermis, stomach, Y-organ, mandibular organ, gill, and gonad). After being treated with DNase I (Fermentas) at 37°C for 30 min, 1 μg total RNA was reverse-transcribed with primer oligo-dT using a RevertAid First Strand cDNA Synthesis Kit (Fermentas). Tissues expression profiles were examined with a semi-quantitative PCR assay. *Arginine kinase* (*AK*, GenBank accession number: JQ031765), a housekeeping gene, was used as an internal control. The PCR was carried out with primer pairs CFSH-QF/-QR and AK-QF/-QR (Supplementary Table 1), respectively, as the following conditions: 94°C for 3 min, 35 cycles of 94°C for 30 s, 58°C for 30 s, and 72°C for 30 s, final extension 72°C for 10 min. PCR products were examined by 1.0% agarose gel and visualized by using ethidium bromide (EB).

### Localization of *Sp-CFSH* by mRNA *in Situ* Hybridization

For mRNA *in situ* hybridization, a pair of gene-specific primer, CFSH-IF and CFSH-IR (Supplementary Table 1), was used for the generation of antisense and sense RNA probes. Digoxigenin (DIG) labeled RNA probes were synthesized via the T7 RNA polymerase (Roche). Based on the tissue expression profiles of *Sp-CFSH*, eyestalk ganglia from male *S. paramamosain* were dissected, and then fixed in 4% paraformaldehyde. After serial dehydration and other treatments, fixed tissues were embedded in paraffin and then sectioned with a thickness of 7 μm, which were prepared for hybridization using the protocol as stated by Shechter et al. (2005). Hybridization was carried out according to the manufacturer’s protocol of DIG RNA Labeling Kit (SP6/T7, Roche, Switzerland).

### Expression and Purification of Recombinant *Sp-CFSH* Protein

The fragment encoding the mature hormone of *Sp-*CFSH was obtained by PCR with primers CFSH-EF and CFSH-ER (Supplementary Table 1) containing two restriction enzyme sites, *BamH I* and *Spe I*, and then sub-cloned into the prokaryotic expression vector pET-His. Subsequently, the recombinant plasmid generated was introduced into the host BL21 (DE3) *E. coli* by transformation for protein expression. Transformed cells were cultured at 37°C in LB medium with 50 μg/ml ampicillin. When OD_600_ reached to 0.6, isopropyl-β-D-thiogalactoside (IPTG) was added to a final concentration of 0.8 mM and cells were cultured for another 8 h at 18°C for protein expression. After harvest by centrifugation, cell pellets were resuspended in binding buffer (20 mM sodium phosphate, 500 mM NaCl, and 20 mM imidazole; pH 8.0) and dissociated by ultrasonic wave. The soluble fraction and insoluble fraction were separated by centrifugation at 12,000 × *g* for 15 min at 4°C and analyzed by 15% SDS–PAGE. The precipitate was harvested and resuspended in binding buffer containing 8 M urea, loaded on an Ni-NTA HisTrap^TM^ FF crude column (GE Healthcare) according to the manufacturer’s instructions, and then washed with 10 ml of the elution buffer (20 mM sodium phosphate, 500 mM NaCl, and 8 M urea) containing 50 mM imidazole, 10 ml of the elution buffer containing 100 mM imidazole, and 5 ml of the elution buffer containing 200 mM imidazole in order. The rCFSH was eluted with 6 ml of the elution buffer containing 300 mM imidazole. Eluting solution were collected and examined by 15% SDS–PAGE. After that, purified rCFSH was renatured using the measure of graded urea dialysis and confirmed by Western blotting. For immunoblotting analysis, the electrophoresed proteins were transferred to a PVDF membrane and analyzed as previously described by Imjongjirak et al. (2005). Finally, the renatured protein was stored at -20°C until use.

### Effect of Recombinant *Sp*-*CFSH* on *Sp-IAG* Expression in Androgenic Glands

The biological activity of rCFSH for inhibiting AG was determined by an *in vitro* experiment. The male crabs (body weight: 168.5 ± 3.5 g; carapace width: 10.3 ± 0.5 cm) were anesthetized on ice for 10 min and sterilized in 75% ethanol for 10 min. AG which is attached to spermaduct were immediately dissected and washed gently by crab saline with antibiotics penicillin G (300 IU/ml) and streptomycin (300 mg/ml) for five times. The AGs were placed in a 24-well cultured plate with 400 μl L15 medium containing antibiotics right after being separated from spermaduct and pre-cultured for an hour. The experiment was divided into two groups: with rCFSH at a final concentration of 10^−6^ M as experimental group and without rCFSH as control group, each group has four repeats (*n* = 4). Finally, AG samples were incubated for another 12 h and RNA was isolated for qRT-PCR assay.

### Quantitative Real-Time PCR

The qRT-PCR was used to determine the relative expression of *Sp-CFSH* in the eyestalk ganglion and *Sp-IAG* in the AG, respectively. The cDNA samples were generated using TransScript^®^ II One-Step gDNA Removal and cDNA Synthesis SuperMix (TransGen Biotech) according to the manufacturer’s protocol. 1 μg total RNA was reverse-transcribed with random primer and the cDNAs generated were diluted four times for qRT-PCR analysis. The reaction system of the qRT-PCR in this study was 20 μl in volume containing 10 μl 2× PCR Master Mix with SYBR Green, 2 μl dilute cDNA, 0.8 μl each primer (1 mM), and 6.5 μl water. The reaction was performed with 7500 Fast Real-Time PCR (Applied Biosystems) under the following conditions: 94°C for 10 min followed by 40 cycles of 94°C for 30 s, 58°C for 30 s, 72°C for 30 s, and a final extension 72°C for 10 min. The reactions were completed in triplicate and normalized to the internal control gene *AK*. Primers used for qRT-PCR were all listed in Supplementary Table 1.

### Statistical Analysis

We calculated the qRT-PCR data using the 2^−ΔΔCt^ method, and then performed statistical analysis. Levene’s test was used for homoscedasticity and statistical significance of the data (*P* < 0.05) was determined using one-way analysis of variance (ANOVA) with post Duncan multiple comparison test (SPSS 20.0). The results were presented as mean ± SEM.

## Results

### Full Length of *Sp-CFSH*

**FIGURE 1:**
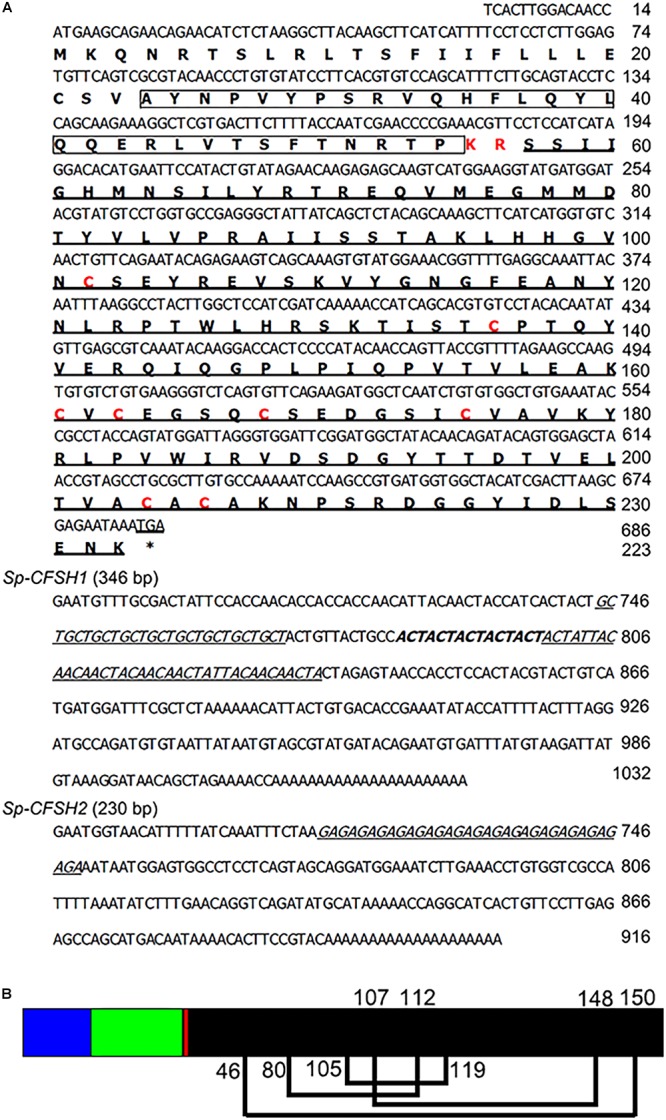
**(A)** The nucleotide and deduced amino acid sequence of *Sp-CFSH1* and *Sp-CFSH2* from the mud crab *S. paramamosain*. The ORF is shown in a single letter code below the nucleotide sequence. The CFSH precursor-related peptide is boxed, the dibasic cleavage site (KR) and eight cysteine residues are indicated in red. The mature peptide and the microsatellites in the 3′-UTR are underlined. **(B)** A schematic diagram of prepro-CFSH. Signal peptide (blue bar), CFSH precursor-related peptide (green bar), a dibasic cleavage site KR (red bar), and the mature hormone (black bar). The eight cysteine residues are predicted to form four putative disulfide bridges connected with lines (C46-150, C80-112, C105-119, and C107-148).

**FIGURE 2:**
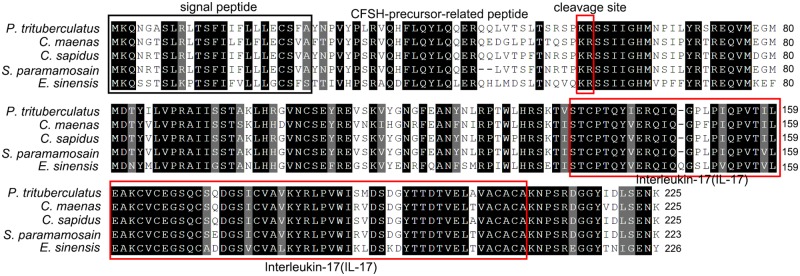
Sequence alignment of deduced amino acid sequences of CFSH cDNAs of brachyuran crabs. Note the conservation of CFSH in brachyuran crabs. Conserved residues are indicated by the black highlights. The signal peptide is highlighted by a black box. The cleavage site (KR) and the domain IL-17 are boxed in red.

Two transcripts of *CFSH*, named *Sp-CFSH1* (GenBank accession number: MF489232) and *Sp-CFSH2* (GenBank accession number: MF489233), were cloned from the eyestalk ganglia of *S. paramamosain* by using PCR with degenerate primers, followed by 5′- and 3′-RACE. It was found that the nucleotide sequences of *Sp-CFSH1* (1032 bp) and *Sp-CFSH2* (916 bp) were identical in 5′-UTR and ORF but different in 3′-UTR (**Figure 1A**), which could be translated into a same hormone (223-aa). The predicted CFSH contained a 23-aa signal peptide, a 33-aa CFSH precursor-related peptide, a dibasic cleavage site (KR), and a 167-aa mature CFSH (**Figure 2**) in order, suggesting that it was synthesized as a preprohormone. There were eight cysteine residues that formed four putative intramolecular disulfide bridges in the mature CFSH: C46-150, C80-112, C105-119, and C107-148 (**Figure 1B**).

### Homology and Phylogenetic Analysis

**FIGURE 3:**
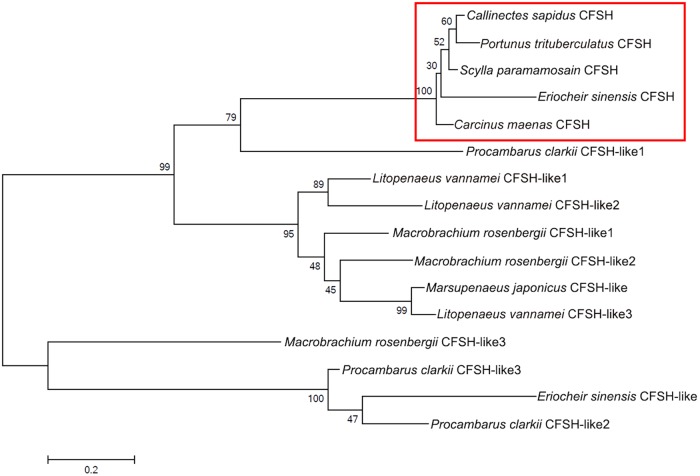
Phylogenetic tree of amino acid sequences of the CFSH in decapod crustaceans. The tree was built by the maximum likelihood (ML) method. *Callinectes sapidus* (GenBank accession number: GU016328.1), *Carcinus maenas* (GenBank accession number: HM594946.1), and the other sequences of CFSH were obtained from (Veenstra, 2016).

The amino acid sequences of *Sp-CFSH* share 95 and 90% identity with that of CFSH in *C. sapidus* and *C. maenas*, respectively. Phylogenetic analysis revealed that CFSH is a highly conserved hormone among brachyuran crabs (**Figures 2**, **3**). The mature hormones of CFSH, as well as CFSH-like, were determined to contain an interleukin-17 (IL-17) domain, indicating that they belonged to a new protein family homologous to the IL-17 family in vertebrates (**Figure 2**). In decapod crustaceans, CFSH can be classified into two types (**Figure 3**).

### Tissue Distribution of *Sp-CFSH* in Males and Females

**FIGURE 4:**
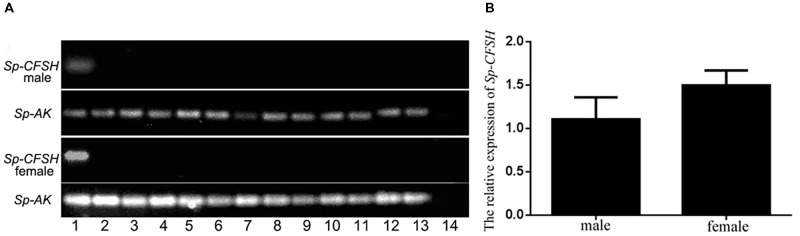
**(A)** Tissue distribution of *Sp-CFSH* in both sexes of prepubertal mud crab *S. paramamosain*. The analysis was generated by PCR assays with cDNAs from various tissues of both sexes and compared with arginine kinase (*AK*) gene. 1, eyestalk ganglion; 2, thoracic ganglion; 3, cerebral ganglion; 4, stomach; 5, ovary or testis; 6, hepatopancreas; 7, heart; 8, hemocytes; 9, muscle; 10, Y-organ; 11, mandibular organ; 12, gill; 13, epidermis; and 14, negative control was shown with the amplification of water. **(B)** The relative expression of *Sp-CFSH* between male and female *S. paramamosain*. Data are shown as means ± SEM of six separate individuals (*n* = 6).

Tissue distribution was determined by PCR with sequence specific primer pairs. The cDNAs of various tissues used for *Sp-CFSH* expression analysis were from prepubertal females and males. This revealed that *Sp-CFSH* was exclusively expressed in the eyestalk ganglia of both sexes of *S. paramamosain* (**Figure 4A**). Unexpectedly, *Sp-CFSH* was found to be highly expressed in the eyestalk ganglia of males, which was not significantly different from that of females (*P* > 0.05) (**Figure 4B**).

### Localization of *Sp-CFSH* by mRNA *in Situ* Hybridization

**FIGURE 5:**
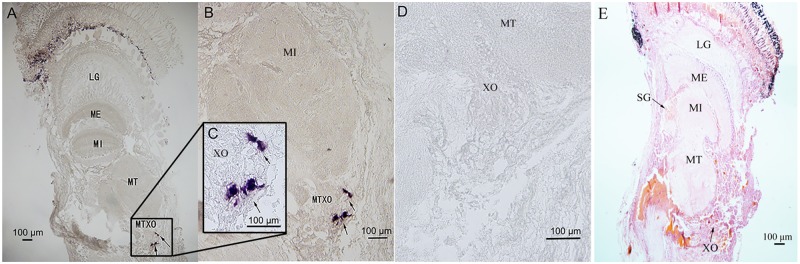
Localization of *Sp-CFSH-*expressing cells in the eyestalk ganglia of male *S. paramamosain*. **(A–C)** Localization of *Sp-CFSH* mRNA by the antisense probe. Arrows indicate the positive signals of *Sp-CFSH*. **(D)** Negative control result with the sense probe. **(E)** Hematoxylin and eosin staining. LG, lamina ganglionaris; ME, medulla externa; MI, medulla interna; MT, medulla terminalis.

The eyestalk ganglia can be divided into lamina ganglionaris (LG), medulla externa (ME), medulla interna (MI), and medulla terminalis (MT) (**Figure 5E**). The X-organ is located at the MT (**Figure 5E**). The antisense *Sp-CFSH* probers gave a clear signal in cytoplasm of neuroendocrine cells in the X-organ of male (**Figures 5A–C**). No signal was observed when a sense probe was used as a negative control (**Figure 5D**).

### The Expression Profiles of *Sp-CFSH* and *Sp-IAG* During Androgenic Gland Development

**FIGURE 6:**
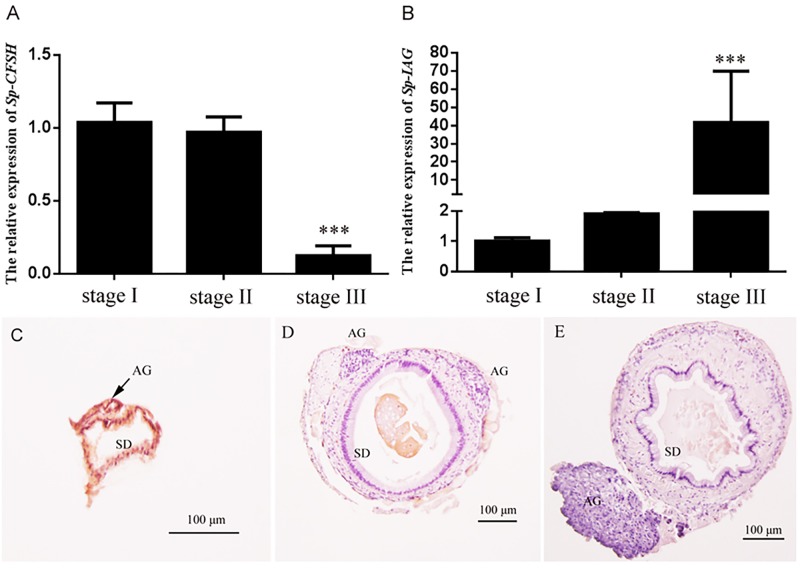
Expression patterns of *Sp-CFSH* **(A)** and *Sp-IAG* **(B)**, and histological characterization of the androgenic gland **(C–E)** at three different development stages of male *S. paramamosain*. Data are shown as means ± SEM of six separate individuals (*n* = 6). Asterisks ^∗∗∗^ (*P* < 0.001) on the top of the error bars indicate highly significant differences from stage I. **(C)** Stage I; **(D)** stage II; **(E)** stage III. SD, sperm duct; AG, androgenic gland.

The expression profiles of *Sp-CFSH* and *Sp-IAG* were demonstrated by qRT-PCR. Based on the definition of AG development (Ye et al., 2003; Liu et al., 2008), *Sp-CFSH* had a high expression at stage I, but decreased gradually from stage II onward, and its expression level was extremely low at stage III (mature stage) of AG development (**Figure 6A**). On the contrary, the level of *Sp-IAG* transcript in the AG increased during the development of AG and reached the peak at stage III, almost 40-fold higher than the level detected at stage I (*P* < 0.001) (**Figure 6B**). This result was in accordance with the histological results that showed the volume of AG, as well as the number of endocrine cells, increased substantially at stage III compared with stage I and stage II (**Figures 6C–E**).

### Expression and Purification of the Recombinant *Sp-CFSH* Protein

The predicted molecular mass of mature CFSH was 18.61 kDa. The rCFSH was expressed with pET-His plasmid as an N-terminal His-tagged fusion protein in the prokaryotic expression system, which increased the molecular weight of the fusion protein to approximately 20 kDa. The whole cell lysate of *E. coli* BL21 with recombinant plasmid, induced with IPTG, in addition to the soluble and insoluble fractions were analyzed by 15% SDS–PAGE (Supplementary Figure 1A). It showed that the recombinant protein of *Sp-CFSH* (∼20 kDa) was expressed after IPTG induction for 8 h. In contrast to the supernatant, the fusion protein was mostly expressed in the precipitate of ultrasonically broken *E. coli* BL21 (DE3). High-purity protein products were obtained after using Ni-NTA column affinity chromatography at 300 mM imidazole concentration (Supplementary Figure 1B). By detecting with prepared anti-His-tag rabbit antibody, the purified recombinant protein, after renaturation, was confirmed by Western blotting; it showed that the antibody could specifically bind to the purified protein (Supplementary Figure 1C). Finally, the renatured protein was obtained by graded urea dialysis.

### Effect of Recombinant *Sp-CFSH* on *Sp-IAG* Expression in AG

**FIGURE 7:**
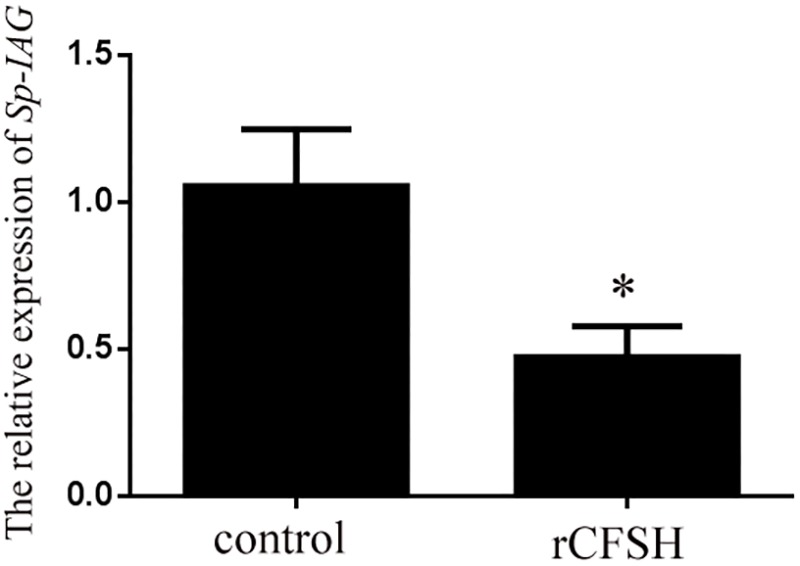
*Sp-IAG* mRNA level in the AG explants after treatment with recombinant *Sp-CFSH* protein (rCFSH). Data are shown as means ± SEM of four separate repeats (*n* = 4). Asterisks (*P* < 0.05) on the top of the error bar indicates significant difference from the control group.

Using the level of *Sp-IAG* transcript as an indicator of AG development, the inhibitory effect of rCFSH on the development of AG was vindicated. The result showed that the *Sp-IAG* transcripts were 60% inhibited when rCFSH was added into the culture medium at a final concentration of 10^−6^ M, which was significantly different from the control (*P* < 0.05) (**Figure 7**).

## Discussion

In crustaceans, studies of sexual differentiation were mainly focused on AG in males (Ventura et al., 2011b). The recent discovery that CFSH was responsible for regulating the development of female phenotypes in the blue crab *C. sapidus* is a major breakthrough (Zmora and Chung, 2013). To date, although the full length of CFSH was merely characterized from two crab species, *C. sapidus* and *C. maenas*, CFSH sequences were identified in several other brachyuran crabs, such as *Portunus trituberculatus* (Veenstra, 2015, 2016) and *Eriocheir sinensis* (Liu et al., 2015; Veenstra, 2016). Moreover, CFSH homologies were also found in shrimps and freshwater crayfish, including *Fenneropenaeus merguiensis* (Powell et al., 2015), *Sagmariasus verreauxi* (Ventura et al., 2014), *Procambarus clarkii* (Veenstra, 2015), *M. rosenbergii* (Suwansa-ard et al., 2015; Veenstra, 2016), and *Litopenaeus vannamei* (Veenstra, 2016). In the present study, we cloned two transcripts of *CFSH*, *Sp-CFSH1* and *Sp-CFSH2*, from the eyestalk ganglion of *S. paramamosain*. The nucleotide sequence of these two transcripts were the same in 5′-UTR and ORF, but different in 3′-UTR (**Figure 1A**). *Sp-CFSH1* (1032 bp) was longer than *Sp-CFSH2* (916 bp) and the identified CFSH in *C. sapidus* (927 bp) (Zmora and Chung, 2013). The difference in the length of 3′-UTR implied potential regulation at the translational level in crustaceans (Manor et al., 2007; Ventura et al., 2009). Alternative gene splicing might be responsible for the presence of two transcripts of *CFSH* in *S. paramamosain.* This phenomenon is similar to the findings of IAGs in *C. sapidus* and *F. chinensis* (Li et al., 2012; Chung, 2014). Microsatellite repeat sequences were found in the 3′-UTR of both *Sp-CFSH1* and *Sp-CFSH*2 (**Figure 1A**). It has been reported that microsatellites in the 3′-UTRs can cause transcription slippage and influence the stability of mRNAs (Ruggiero et al., 2003; Li et al., 2004). However, the functional importance of these repeats is unknown and varies depending on the species (Li et al., 2004). It would be interesting to understand the precise regulation of these microsatellites in 3′-UTR of *Sp-CFSH*. Clearly it warrants further research.

Crustacean female sex hormone homologies were firstly named by Veenstra (2016), which were classified into two types. Here we reanalyzed these sequences and subsequently renamed the homologous proteins as CFSH-like, based on the IL-17 domain. Interestingly, another CFSH-like protein was found in *E. sinensis* and three CFSH-like proteins were found in coexistence in *P. clarkii*, *M. rosenbergii*, and *L. vannamei*, respectively (**Figure 3**). It appears common that this gene have several paralogs in crustacean species (Veenstra, 2016). The primary structure of CFSH and CFSH-like is conserved, which all contain a precursor-related peptide and an IL-17 domain in the mature hormone. The identification of the IL-17 domain in the mature hormone suggests that a new protein family homology to the IL-17 family may exist in crustaceans. Up to now, the function of this new protein family in crustaceans is unknown except CFSH. In vertebrates, the IL-17 family is a group of potent proinflammatory cytokines produced by the activated memory T cells (Shabgah et al., 2014; Kumar et al., 2016). It has been reported that the IL-17 signaling pathway is involved in the innate and adaptive immunity of vertebrates. Recently, the involvement of IL-17 in immunity has also been documented in some invertebrates such as pearl oyster *Pinctada fucata* (Wu et al., 2013). Furthermore, cytokines, also known as immunoregulatory proteins, have been shown to affect the neuroendocrine events of reproduction, e.g., ovarian/testicular function (Ben-Rafael and Orvieto, 1992; Rutanen, 1993). It has been reported that IL-1 can stimulate testosterone secretion in adult male rat Leydig cells *in vitro* (Warren et al., 1990). Although structural similarity does not necessarily imply functional similarity, the IL-17 signaling pathway provides a clue to understand the CFSH signaling system.

In crustaceans, the X-organ appeared to be the only site of CFSH synthesis. In *S. paramamosain*, our result showed that *Sp-CFSH* was exclusively expressed in the eyestalk ganglion. This is the same as the previous study of *C. sapidus*, in which CFSH was specifically present in the X-organ-sinus gland of the eyestalk ganglion (Zmora and Chung, 2013). Similarly in *S. verreauxi* and *F. merguiensis*, CFSH-like was uniquely expressed in the eyestalk ganglion (Ventura et al., 2014; Powell et al., 2015). CFSH eyestalk specificity was further confirmed by the result of *in situ* hybridization of the present study, which showed that *Sp-CFSH* mRNA was localized in the neuroendocrine cells of X-organ (**Figure 5**), a result in agreement with the findings of an immunohistology study in *C. sapidus* by Zmora and Chung (2013). The eyestalk specificity of CFSH might explain observation in several crustaceans, such as the spider crab *Libinia emarginata* (Saigusa et al., 2002) and the blue crab *C. sapidus* (Zmora and Chung, 2013). Female eyestalk ablation at the prepuberty stage led to impaired mating and brooding systems.

Our result showed that *Sp-CFSH* was highly expressed in prepubertal male *S. paramamosain*, which was not significantly different from the levels found in females (**Figure 4B**). In *S. verreauxi*, *CFSH-like* was also found to be expressed in both male and female eyestalk ganglia at roughly same levels by comparative transcriptome analysis (Ventura et al., 2014). These results contradicted an earlier study that suggested CFSH was female specific: CFSH was reported to have a high expression in the eyestalk ganglia of adult females, but was extremely low in adult males in *C. sapidus*. Moreover, CFSH proteins were only isolated from female sinus gland extracts (Zmora and Chung, 2013). The difference probably was related to different development stages of crabs used in tissue distribution analyses in the two studies. As observed in this study, the level of *Sp-CFSH* was high in the prepubertal males but also extremely low in mature males (**Figure 6A**). In fact, the low expression in mature males, in our experiment, was similar to that of the adult male *C. sapidus* (Zmora and Chung, 2013). Unfortunately, the expression of *CFSH* in prepubertal *C. sapidus* males was not studied, so no comparison could be made. Nevertheless, our result clearly demonstrated that CFSH is not a female specific hormone.

AG and/or IAG have been shown to play a crucial role in regulating male sex differentiation. For example, in *M. rosenbergii*, the male morphotypic differentiation and gonad activity was positively related to the level of *Mr-IAG* (Ventura et al., 2011a), and the change of *Mr-IAG* transcript level was closely correlated with the development of AG (Phoungpetchara et al., 2011). In addition, AG ablation could turn males into neo-females (Aflalo et al., 2006), and the silencing of *Mr-IAG* in inmature males impeded spermatogenesis and the development of male secondary sexual characteristics (Ventura et al., 2009). In the present study, it was also revealed that both the volume of AG and the amount of endocrine cells increased substantially at stage III when compared to stages I and II (**Figures 6C–E**); correspondingly, the level of *Sp-IAG* transcripts rose sharply at stage III (*P* < 0.001) (**Figure 6B**).

In crustacean males, it is well known that AG is under the inhibitory control of the eyestalk ganglion, because eyestalk ablation can result in hypertrophy (Sroyraya et al., 2010). The AG inhibiting factor(s) produced by the eyestalk ganglion might be diverse. In the freshwater prawn *Macrobrachium nipponense*, it was reported that both gonad-inhibiting hormone (GIH) and molt-inhibiting hormone (MIH) had the inhibitory effects on *IAG* gene expression (Li et al., 2015). In *S. paramamosain*, eyestalk ablation of males (body weight, 150.5 ± 20.3 g) led to the up-regulation of *Sp-IAG* expression (Zhang et al., 2014), suggesting that *Sp-IAG* was also negatively regulated by the eyestalk-derived factor(s). In this study, *Sp-CFSH* was found to have the opposite expression tendency compared to *Sp-IAG* during the development of AG. That is, at the early stage, high expression of *Sp-CFSH* transcripts was detected in eyestalk ganglion (**Figure 6A**) whereas the expression of *Sp-IAG* transcripts was low in AG (**Figure 6B**). Therefore, we hypothesized that CFSH might suppress the development of AG at this early stage. To verify this, the recombinant CFSH (rCFSH) was obtained to assay its bioactivity *in vitro* and the result showed that the *Sp-IAG* expression transcripts in AG were reduced by about 60% by rCFSH, at a concentration of 10^-6^ M (**Figure 7**). Such a result demonstrates that CFSH generates an inhibitory effect on AG activity. Hence, our results suggest that CFSH might regulate male sex differentiation via AG.

Our result also showed that at the mature stage (stage III) of male *S. paramamosain*, the expression of *Sp-CFSH* was extremely low (**Figure 6A**), while the level of *Sp-IAG* transcript was high (**Figure 6B**). A possible explanation for this phenomenon could be that AG/IAG affected the expression of *Sp-CFSH* in eyestalk ganglion in reverse. In the red claw crayfish *Cherax quadricarinatus*, implantation of AG into immature females caused the inhibition of female secondary sex characteristics (Khalaila et al., 2001; Manor et al., 2004), especially the maternal brooding-related characteristics, namely the relative abdomen widths and the number of simple setae were significantly reduced (Barki et al., 2003); it is now known that the maternal brooding-related characteristics were largely controlled by CFSH (Zmora and Chung, 2013). Clearly, such an explanation is largely speculative, further proposed designed experiments, such as *in vivo* bioassay that can confirm whether AG/IAG impacts upon *CFSH* expression, are needed to test this hypothesis.

In summary, our study characterized two transcripts of *Sp-CFSH* from the eyestalk ganglion of the mud crab *S. paramamosain*. It appears for the first time that CFSH has been reported to be highly expressed in male crustaceans; and the inhibitory effect of CFSH on AG has been demonstrated. Furthermore, our research suggests the existence of putative antagonism between CFSH and IAG in male *S. paramamosain*, which warrants future investigation.

## Author Contributions

AL carried out the majority of the experiments and prepared the manuscript. JL and FL were involved in cDNA cloning, hybridization *in situ*, and statistical analysis. YH was involved in histological analysis, qRT-PCR analysis, data analysis, and GW was involved in the data analysis and preparation of the manuscript. HY designed the experiments, analyzed the results, and prepared the manuscript.

## Conflict of Interest Statement

The authors declare that the research was conducted in the absence of any commercial or financial relationships that could be construed as a potential conflict of interest.

## Footnotes

1 http://www.ncbi.nlm.nih.gov/gorf/gorf.html

2 http://www.cbs.dtu.dk/services/SignalP/
