# Supplementary material for: Crustacean Female Sex Hormone From the Mud Crab Scylla paramamosain Is Highly Expressed in Prepubertal Males and Inhibits the Development of Androgenic Gland
Source: Front Physiol. 2018 Jul 17;9:924. doi: 10.3389/fphys.2018.00924 (PMC6056722; doi:10.3389/fphys.2018.00924)
Supplement: Supplementary file 1 [file Table_1.DOCX]

| **Supplemental Table 1.** Summary of primers used in this study | | | |
| --- | --- | --- | --- |
| **Primer(5’-3’)** | | **Primer sequence** | **Application** |
| CFSH-DF | CCGTACAGAACAAGAGAGCAAGTC | | cDNA cloning |
| CFSH-DR | TTYTTGGCACARTCGCAGGCTACRG | | cDNA cloning |
| CFSH-3F1 | CTGGTGCCGAGGGCTATTATC | | 3’RACE |
| CFSH-3F2 | ATCTGTGTGGCTGTGAAATACCG | | 3’RACE |
| CFSH-5R1 | CATCCGAATCCACCCTAATCC | | 5’RACE |
| CFSH-5R2 | GTTGTATGGGGAGTGGTCCTTGT | | 5’RACE |
| CFSH-ISHF | CCATCGATCAAAAACCATCAGCACG | | *in situ* hybridization |
| CFSH-ISHR | CCATCACGGCTTGGATTTTTGGCAC | | *in situ* hybridization |
| CFSH-EF | CGCGGATCCTCCTCCATCATAGGACACATGAATTC | | CFSH expression |
| CFSH-ER | GGACTAGTTTTATTCTCGCTTAAGTCGATGTAG | | CFSH expression |
| CFSH-QF | CGTGTCCAGCATTTCTTGCAGTACC | | qRT-PCR |
| CFSH-QR | TCATGTGTCCTATGATGGAGGAACG | | qRT-PCR |
| IAG-QF | ATCCTTTTCCTCCGTTTGCC | | qRT-PCR |
| IAG-QR | TCGGGTCTTCGTCTTGTTCC | | qRT-PCR |
| AK-QF | TTCCTCCACCCTGTCCAACC | | qRT-PCR |
| AK-QR | GAAGCGGTCACCCTCCTTGA | | qRT-PCR |


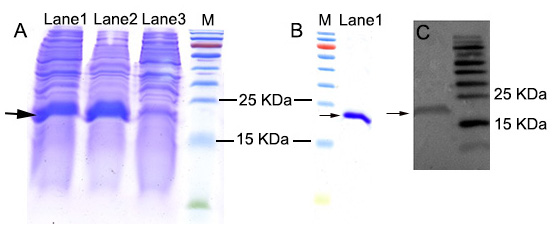


**Supplemental Fig. 1.** Expression and purification of rCFSH expressed in bacterial cell lysates, as analyzed by 15 % SDS-PAGE. (A) Lane 1: cell lysate with IPTG induction; Lane 2: insoluble fraction of the cell lysate; Lane 3: soluble fraction of the cell lysate. (B) M: marker; Lane 1: isolated protein products (*Sp-*CFSH) after Ni column purification and renaturation. (C). Western blotting analysis of the rCFSH after purification and renaturation. Arrows indicate the target protein product.
